# Supplementary material for: Effect of circadian rhythm disruption on benign prostatic hyperplasia in rats
Source: PeerJ. 2025 Oct 15;13:e20173. doi: 10.7717/peerj.20173 (PMC12535237; doi:10.7717/peerj.20173)
Supplement: Supplemental Information 6 [file peerj-13-20173-s006.pdf]

The effect of circadian rhythm disorder on benign prostatic hyperplasia in rats

Xiaoxue Huang[1], Xiaohu Tang[2], Yuanzhao Xu[2,3], Zhiyan Liu[4], Guangheng Luo[2*]

*:Correspondence to luoguangheng1975@126.com

The PDF File includes:

Figs.S1 to S4

Table S1

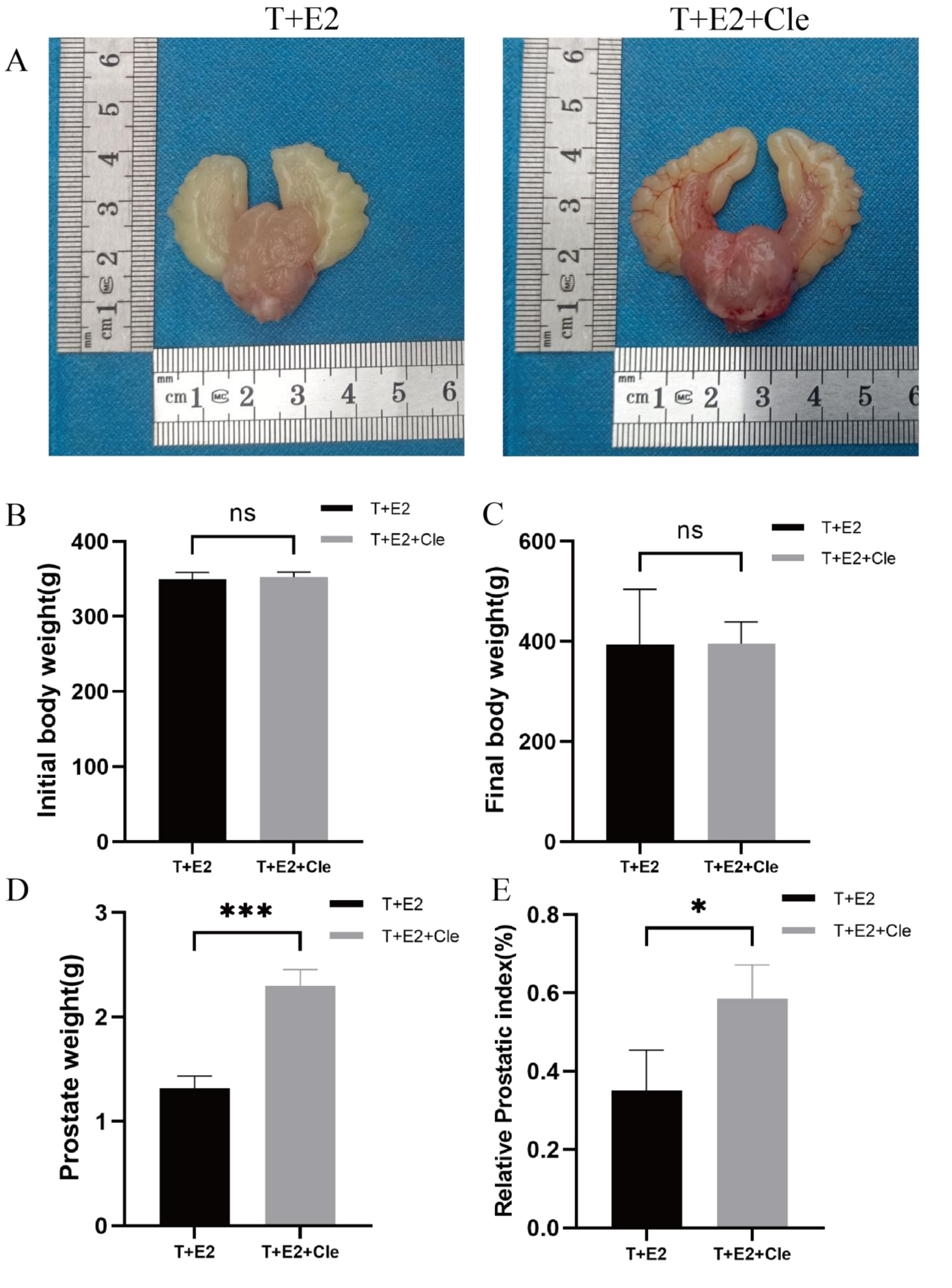

Figure S1 Analysis of prostate indexes in rats after circadian rhythm disorder intervention.

(A) The morphology of the prostate of rats in each group. (B) Initial body weight of rats in each group. (C)Final body weight of rats in each group. (D) prostate weights of rats in each group. (E) PI values of rats in each group. $*p < 0.05$, $**p < 0.01$, $***p < 0.001$, ns, not significant, when compared with the T+E2 group. n=3

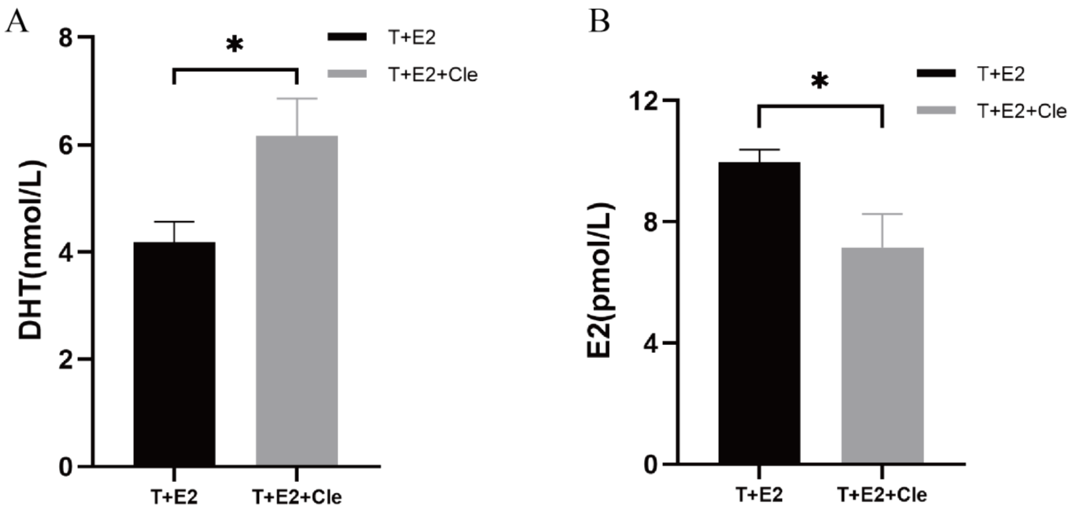

Figure S2. Assessment of serum DHT and E2

(A) Levels of DHT in serum of rats in various groups detected by ELISA. (B) Levels of E2 in serum of rats in various groups detected by ELISA. Each bar in the graph represents the mean ± S.D. *$p < 0.05$ compared with T+E2 group. n = 3

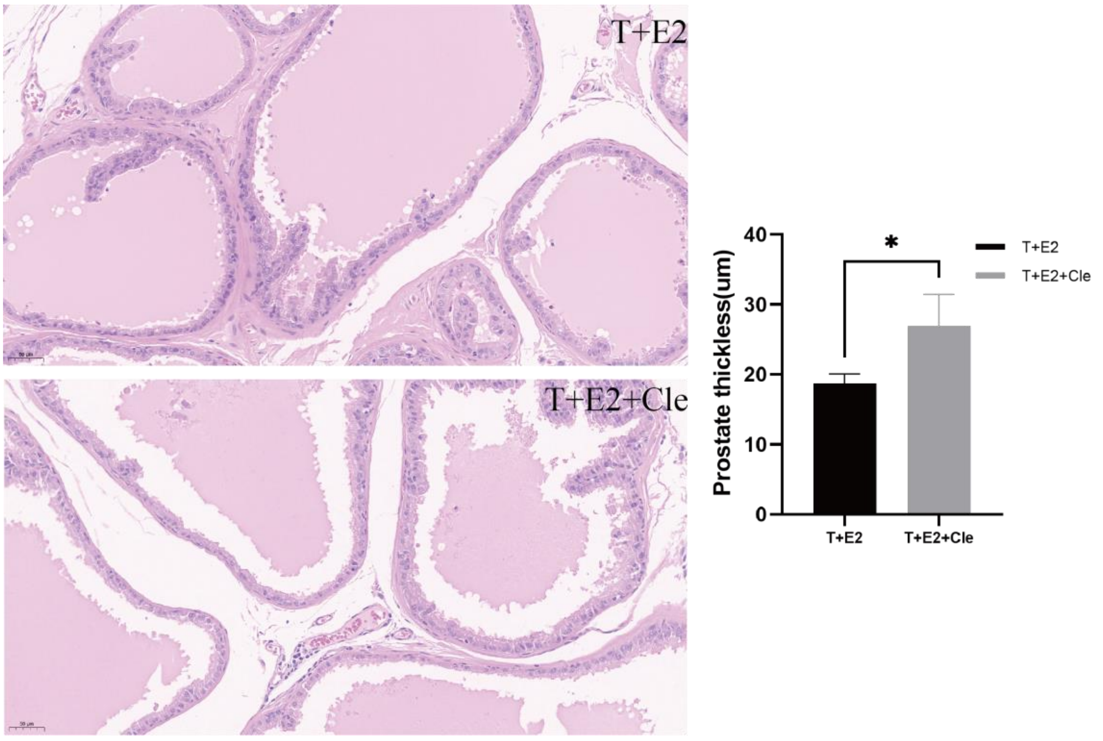

Figure S3. Histological morphological features

H&E staining for pathological changes of rats' prostate tissues (left panel), and the prostate thickness of rats (right panel). Each bar in the graph represents the mean ± S.D. Scale bar=50μm, n=3. *$p < 0.05$, when compared with the T+E2 group.

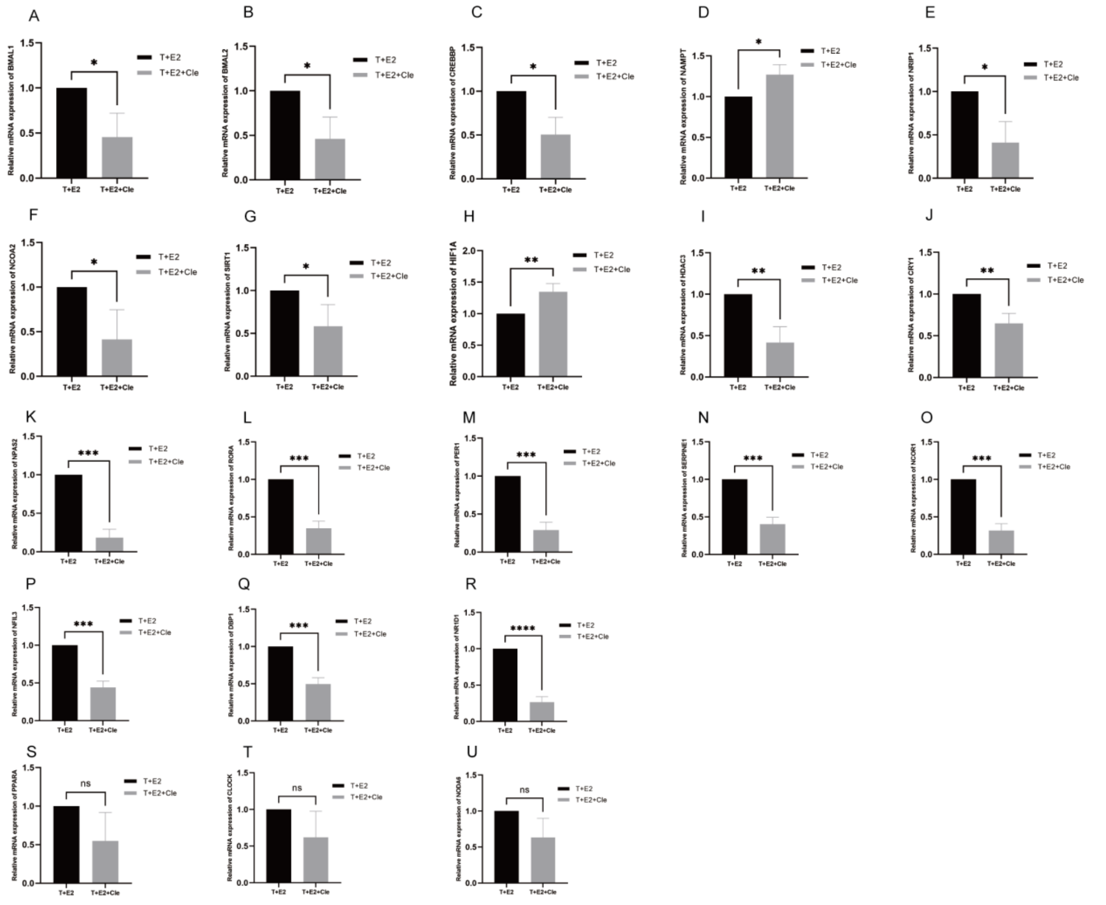

Figure S4. qRT - PCR verify the relative mRNA expression of the key 21 circadian genes.

Effect of T+E2+Cle and T+E2 at the level of relative mRNA expression of circadian genes:(A) BMAL1, (B) BMAL2, (C) CREBBP, (D) NAMPT, (E) NR1P1, (F) NCOA2, (G) SIRT1, (H) HIF1A,(I) HDAC3, (J) CRY1, (K) NPAS2, (L) RORA, (M) PER1, (N) SERPINE1, (O) NCOR1, (P) NFIL3, (Q)DBP, (R) NR1D1, (S) PPARA, (T) CLOCK, (U) NODA6. T-tests were used for statistical analysis.*$p<0.05$, **$p<0.01$, ***$p<0.001$, ****$p<0.0001$, compared to the T + E2 group.

Table S Relative gene expression levels and their statistical differences

Up-regulated genes	Down-regulated genes	P＜0.05	P＞0.05
NAMPT	NCOA2	NAMPT	CLOCK
HIF1A	CREBBP	NCOA2	NODA6
	BMAL1	NRIP1	PPARA
	NRIP1	SIRT1	
	SERP1NE1	RORA	
	NCOR1	NR1D1	
	DBP1	CREBBP	
	RORA	BMAL1	
	NR1D1	BMAL2	
	BMAL2	HIF1A	
	CRY1	HDAC3	
	PER1	NPAS2	
	HDAC3	NFIL3	
	SIRT1	SERP1NE1	
	NFIL3	NCOR1; PER1	
	NPAS2	DBP1; CRY1	
